# Real-World Effectiveness of AI-Enabled Clinical Decision Support in Primary Care: Systematic Review

**DOI:** 10.2196/98630

**Published:** 2026-10-08

**Authors:** Yash Jain, Dishan Wu, Zhong Wang

**Affiliations:** 1 Department of General Practice, Beijing Tsinghua Changgung Hospital School of Clinical Medicine, Tsinghua Medicine Tsinghua University Beijing, Beijing China

**Keywords:** artificial intelligence, AI, clinical decision support systems, machine learning, primary health care, primary care, systematic review, implementation science

## Abstract

**Background:**

AI-enabled clinical decision support systems (CDSS) are being used in primary care, but their effects on clinical decisions and patient outcomes remain uncertain.

**Objective:**

This study aimed to synthesize real-world evaluations of clinician-facing AI-enabled CDSS in primary care and assess evidence for diagnostic performance, changes in care, clinician efficiency, and patient-important outcomes.

**Methods:**

Following PRISMA (Preferred Reporting Items for Systematic Reviews and Meta-Analyses) 2020, 5 principal bibliographic databases (PubMed, Scopus, Web of Science Core Collection, Cochrane CENTRAL, and CINAHL Ultimate), a supplementary EBSCOhost platform search, and 2 trial registries were searched for publications from 2011 through 2025. Eligible studies evaluated a learned (data-derived) or case-based AI component used by clinicians in routine primary or ambulatory care and reported a clinical, behavioral, process, efficiency, or diagnostic accuracy outcome. Screening was performed independently by 2 reviewers. Risk of bias was assessed with design-appropriate tools, and GRADE (Grading of Recommendations Assessment, Development and Evaluation) was applied to 4 outcome bodies. Deterministic rule-based tools were retained only as contextual comparison.

**Results:**

A total of 66 reports were assessed at full text, and 24 studies met the review criteria; 10 were AI-enabled and formed the appraised review population, while 14 deterministic studies were contextual only. Detection findings were inconsistent: AI electrocardiography increased new low ejection fraction diagnoses (odds ratio [OR] 1.32, 95% CI 1.01-1.61), whereas an electronic health record machine learning dementia marker used alone did not (adjusted OR 0.84, 95% CI 0.63-1.11). Diagnostic studies showed high melanoma discrimination (area under the receiver operating characteristic curve [AUROC] 0.960) but only moderate glaucoma discrimination (AUROC 0.80; sensitivity 65%). Nonrandomized before-after studies reported increased urinary tract infection treatment success (from 75% to 80%) and changed diabetic retinopathy screening criteria in 3 of 4 general practitioners. A pre-exposure prophylaxis trial was null overall; a multicomponent fall prevention CDSS improved shared decision-making, with inconclusive medication-change effects. Clinicians reported lower estimated record review time in asthma care. Neither trial assessing patient-important outcomes demonstrated benefit from AI-CDSS; one musculoskeletal functional outcome favored usual care.

**Conclusions:**

Evidence published through December 2025 showed mixed effects of AI-enabled CDSS in primary care. Some studies reported improvements in detection or care processes, but patient-important benefit was not demonstrated. Heterogeneity and limited outcome measurement prevented causal explanations for these differences. Future trials should assess clinical decisions and patient outcomes together.

**Trial Registration:**

PROSPERO CRD420261302104; https://www.crd.york.ac.uk/PROSPERO/view/CRD420261302104

## Introduction

AI-enabled clinical decision support systems (CDSS) can provide predictions or recommendations during primary care consultations. Their clinical value depends on whether they improve decisions and outcomes in routine care. Diagnostic accuracy, changes in management, clinician workload, and patient-important outcomes therefore require separate evaluation.

Susanto et al [1] reviewed machine learning CDSS, predominantly in secondary and tertiary care, and found mixed effects on decision-making, care delivery, and patient outcomes. Gomez-Cabello et al [2] described heterogeneous AI-CDSS implementations in primary care. These reviews provide a basis for examining primary care effectiveness while distinguishing systems with learned components from deterministic decision rules.

This review assesses real-world evaluations of clinician-facing AI-enabled CDSS in primary care. We distinguish diagnostic and case-finding performance, changes in clinical decisions or care processes, clinician efficiency, and patient-important outcomes. The support-impact-outcome framework organizes these outcome levels without assuming that an improvement at one level causes improvement at another.

## Methods

### Overview

We followed the PRISMA (Preferred Reporting Items for Systematic Reviews and Meta-Analyses) 2020 statement [3]. The completed checklist is provided in Multimedia Appendix 1 [4-13]. The protocol was registered with PROSPERO (CRD420261302104). Registration timing and subsequent changes to the methods are described in Multimedia Appendix 1 [4-13].

### Operational Definition of AI

AI-enabled systems were defined as those in which a clinically relevant prediction, classification, retrieval, or recommendation depended on a data-derived model, including machine learning, deep learning, natural language processing, or case-based reasoning that generated patient-specific output from prior cases. Multicomponent CDSS were eligible when a learned component formed part of the deployed intervention; effects were attributed to the whole intervention when that component could not be isolated. Deterministic alerts, guideline rules, electronic reminders, and fixed clinical prediction equations were classified separately. Studies of these tools were retained as contextual evidence and were neither formally appraised nor used to support conclusions about AI effectiveness.

### Inclusion Criteria

Studies were eligible when they (1) evaluated a clinician-facing CDSS used during routine primary or ambulatory care; (2) included a learned (data-derived) or case-based AI component under the operational definition above; (3) involved licensed clinicians delivering first-contact or continuing primary care, including general practitioners (GPs), family physicians, primary care pediatricians, and allied health professionals such as primary care physiotherapists; (4) evaluated real patient care rather than simulated cases; and (5) reported an evaluable diagnostic accuracy, clinical, behavioral, care process, efficiency, or patient-important outcome against a comparator or reference standard. Deterministic studies meeting the other criteria were retained only for the contextual stream.

### Exclusion Criteria

We excluded studies that did not evaluate clinician-facing use in real primary or ambulatory care, including simulations or vignettes, retrospective reader or benchmark studies, model development or validation studies without prospective clinical use, patient-facing tools without clinician-facing decision support, and specialist or hospital-only settings. We also excluded protocols; conference abstracts without a full evaluation; and feasibility-, usability-, or attitude-only reports without an evaluable clinical or care-process outcome. Uncontrolled reports were excluded when no comparator or reference standard allowed an effect estimate.

### Information Sources and Search

Five principal bibliographic databases were searched: PubMed, Scopus, Web of Science Core Collection, Cochrane CENTRAL, and CINAHL Ultimate. A supplementary search was run across 10 institutionally available EBSCOhost databases; EBSCOhost is treated as a platform rather than as a database. ClinicalTrials.gov and the World Health Organization (WHO) International Clinical Trials Registry Platform (ICTRP) were also searched for registered and unpublished evaluations. The registered PROSPERO protocol specifies a publication window of January 1, 2011, through December 31, 2025. The upper bound defines a fixed, complete calendar-year publication window; the later search dates allowed retrieval of records indexed after that boundary. No language limit was applied at the search stage; the registered protocol specifies no language restrictions on eligibility.

The search used 3 concepts: AI, clinical decision support, and primary or ambulatory care, with database-specific controlled vocabulary and free text. Trial registries were searched more broadly because registry records contain less standardized descriptive information. Exact executed strings, quotation behavior, filters, search dates, and yields are reproduced in Multimedia Appendix 1 [4-13].

### Study Selection and Data Extraction

Titles and abstracts and, subsequently, full-text reports were assessed independently by 2 reviewers. Disagreements were resolved by discussion and, where necessary, adjudication by the senior author. Records lacking an abstract were screened using the title, subject headings, indexing terms, and available record metadata rather than requiring all 3 search concepts to appear in the visible citation. Data extraction captured setting, condition, model class, comparator or reference standard, outcome type, effect estimate, and reported uptake or fidelity. One reviewer extracted the data and a second reviewer independently checked each field against the primary report. Model class assignment and outcome domain coding were performed independently by 2 reviewers using the definitions above, with senior adjudication for borderline systems.

### Risk of Bias and Certainty of Evidence

Risk of bias was assessed with the standard Cochrane risk of bias 2 (RoB 2) tool for the individually randomized trial [14], the RoB 2 cluster-randomized extension for the 5 cluster trials [15], ROBINS-I (Risk of Bias in Non-Randomized Studies of Interventions) for nonrandomized intervention studies [16], and QUADAS-2 (Quality Assessment of Diagnostic Accuracy Studies 2) for diagnostic accuracy studies [17]. QUADAS-2 risk of bias and applicability were reported separately. GRADE (Grading of Recommendations Assessment, Development and Evaluation) [18] was applied to diagnostic case finding, diagnostic accuracy and screening, clinician efficiency, and patient-important clinical outcomes. Each rating addresses the clinical inference stated in the summary of findings within the evaluated settings; it does not represent a pooled effect across diseases. Differences in populations, interventions, outcomes, and study methods informed the certainty judgments. Care-process studies were synthesized descriptively because their end points did not support a common effect estimate. No meta-analysis was performed because conditions, interventions, and outcomes differed within each body. Selective reporting was considered in the study-level assessments. Publication bias and small-study effects were not formally assessed because each rated body contained at most 2 studies.

### Ethical Considerations

This review used published aggregate data only and involved no identifiable participant information or intervention. Under the policy of the Department of General Practice, Beijing Tsinghua Changgung Hospital, secondary analysis of published literature, institutional review board review was not required, and no application was submitted.

## Results

### Study Selection

The final searches identified 3586 records from the 5 principal bibliographic databases and the supplementary EBSCOhost platform search: PubMed (n=821), Scopus (n=927), Web of Science (n=634), Cochrane CENTRAL (n=111), CINAHL Ultimate (n=890), and EBSCOhost (n=203). CINAHL Ultimate was searched separately and is not part of the 203-record supplementary EBSCOhost total. Of the EBSCOhost records, 183 were exportable, and 20 were screened manually in the platform. Across the exportable database and platform records, deduplication left 2265 unique records; adding the 20 manually screened EBSCOhost records gave 2285 database and platform records screened. The 2 registries returned 537 records (ClinicalTrials.gov n=433 and WHO ICTRP n=104); 469 records remained after duplicate or overlap removal and were screened separately, with no eligible published evaluation identified. A total of 66 reports were assessed at full text, 42 were excluded with reasons, and 24 studies met the review criteria. In total, 10 used AI-enabled systems and formed the appraised evidence population; 14 used deterministic rule-based or fixed-equation logic and were retained only as contextual comparison. Figure 1 shows the study flow, Table 1 classifies the included studies, and Table 2 summarizes full-text exclusions.

**Figure 1:**
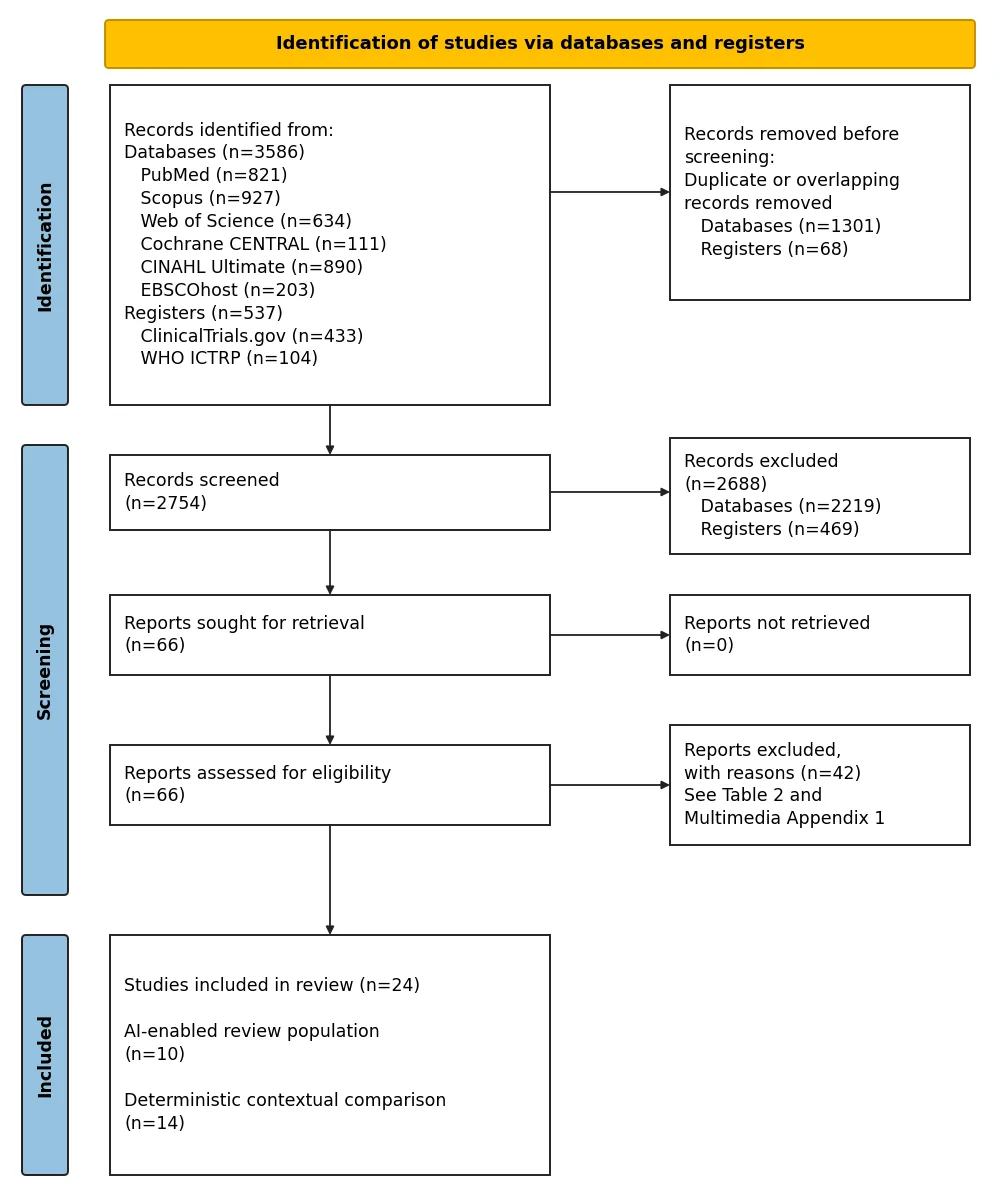
Study selection (PRISMA [Preferred Reporting Items for Systematic Reviews and Meta-Analyses] 2020). Records were identified from 5 principal bibliographic databases, a supplementary EBSCOhost platform search, and 2 trial registries. Twenty EBSCOhost records that could not be exported were screened manually in the platform. Deterministic rule-based and fixed-equation studies that met the other real-world criteria are shown in the included total but are contextual only and are not part of the appraised AI evidence population.

**Table 1:** Classification of the 24 studies meeting the review criteria.

Study	Class^a^	Design	Model or logic	Condition or setting	Outcome domain
Yao et al [4], 2021	AI-enabled	Cluster RCT^b^	Deep CNN^c^ applied to ECG^d^	Low ejection fraction; primary care	Diagnostic case-finding
Boustani et al [5], 2025	AI-enabled	Three-arm cluster RCT	EHR^e^ ML^f^ passive digital marker	Dementia detection; FQHC^g^ primary care	Diagnostic case-finding
Papachristou et al [6], 2024	AI-enabled	Prospective diagnostic accuracy study	Deep learning and computer vision	Melanoma; 36 primary care centers	Diagnostic accuracy
Pinto et al [7], 2025	AI-enabled	Uncontrolled (historical) before-after study	Deep learning diabetic retinopathy screening system (NaIA-RD)	Diabetic retinopathy screening; primary care	Clinician decision and screening process
Jan et al [8], 2025	AI-enabled	Prospective pragmatic diagnostic accuracy study	Deep learning fundus image glaucoma classifier	Glaucoma screening; 2 general practice clinics	Diagnostic accuracy and referral support
Herter et al [9], 2022	AI-enabled	Controlled before-after study	Interpretable ML decision-tree classifiers	Urinary tract infection; primary care	Care process and treatment
Volk et al [10], 2024	AI-enabled	Cluster RCT	EHR ML HIV risk-prediction model	HIV PrEP^h^; primary care	Care process
Westerbeek et al [11], 2025	AI-enabled	Cluster RCT	EHR CDSS^i^ with integrated data-derived fall risk-prediction model plus guideline rules	Medication-related fall prevention; general practice	Shared decision-making and medication process
Seol et al [12], 2021	AI-enabled	Individual RCT	NLP^j^ and ML	Childhood asthma; primary care	Efficiency and patient-important
Granviken et al [13], 2024	AI-enabled	Cluster RCT	Case-based reasoning from a library of prior cases	Musculoskeletal pain; primary care physiotherapy	Patient-important
Rossom et al [19], 2022	Contextual deterministic	Real-world deployment	Rule-based EHR CDSS	Cardiovascular risk; primary care	Process and surrogate
Shi et al [20], 2023	Contextual deterministic	Real-world deployment	Guideline-logic CDSS	Diabetes; team-based primary care	Surrogate outcomes
Cho et al [21], 2023	Contextual deterministic	Real-world deployment	Rule-based EHR prompt	Vitamin D test ordering	Process
Salinas et al [22], 2025	Contextual deterministic	Real-world deployment	Fixed SCORE2^k^ equation	Cardiovascular risk assessment	Process and surrogate
Samal et al [23], 2022	Contextual deterministic	Real-world deployment	Fixed KFRE^l^ equation	Kidney failure risk; primary care	Process
Mohanty et al [24], 2025	Contextual deterministic	Real-world deployment	Passive rule-based CDSS	Liver fibrosis risk; weight management clinic	Engagement and process
Arts et al [25], 2017	Contextual deterministic	Real-world deployment	Rule-based CDSS	Stroke prevention in atrial fibrillation	Process
Marcolino et al [26], 2021	Contextual deterministic	Real-world deployment	Rule-based CDSS	Hypertension and diabetes	Process
Atlas et al [27], 2025	Contextual deterministic	Real-world deployment	Rule-based CDSS	Cervical cancer screening follow-up	Process
Rossom et al [28], 2025	Contextual deterministic	Real-world deployment	Rule-based CDSS	Opioid use disorder; primary care	Process and uptake
Healey et al [29], 2025	Contextual deterministic	Real-world deployment	Digital history-taking and rule-based logic	Ambulatory differential diagnosis	Process
Spann et al [30], 2025	Contextual deterministic	Real-world deployment	Electronic decision aid	Liver disease; primary care	Process and surrogate
Fan et al [31], 2025	Contextual deterministic	Real-world deployment	Knowledge-based system	General primary care	Process and diagnostic
Ru et al [32], 2025	Contextual deterministic	Real-world deployment	Guideline rule engine and fixed risk scores	Atrial fibrillation anticoagulation; primary care	Process

^a^AI-enabled studies contained a learned (data-derived) or case-based component and were formally appraised. Deterministic studies met the other real-world eligibility criteria but were not part of the AI evidence population and were not used to corroborate AI effectiveness.

^b^RCT: randomized controlled trial.

^c^CNN: convolutional neural network.

^d^ECG: electrocardiogram.

^e^EHR: electronic health record.

^f^ML: machine learning.

^g^FQHC: federally qualified health center.

^h^PrEP: pre-exposure prophylaxis.

^i^CDSS: clinical decision support system.

^j^NLP: natural language processing.

^k^SCORE2: Systematic Coronary Risk Evaluation 2.

^l^KFRE: Kidney Failure Risk Equation.

**Table 2:** Reports assessed at full text and excluded, by reason (n=42).^a^

Reason for exclusion	Representative studies	n
Simulation, vignette, or reader study	Susanto 2025; sore-throat ML-CDSS^b^; AI fundus reader; SPIRO-AID; low-back-pain vignette models; OpenEvidence	6
Benchmark against clinicians, no deployment	Simao 2025; DermaAId; Escale-Besa 2023	3
Model development and validation without prospective clinical use	Sunjaya 2025; POLAR Diversion; Medicaid impactability model	3
Not clinician-facing or not a CDSS^c^	selfBACK; telehealth matched cohort; Wang 2019; Zaman 2025; Gerdeskold 2020; Megerian 2022; Cherokee Nation HCV^d^ program; integrated personalized-care model	8
Specialist, secondary, or nonprimary care setting	Epilepsy surgery alerts; Smart Scope; Benrimoh AID-ME; MEDINFO LLM^e^ RCT^f^	4
Prototype, feasibility, or pilot	Myers 2026; COVID home-isolation CDSS; Bilaver iREACH; Jaremko; Krakower PrEP^g^; FIND-AF; retinal photography CVD^h^ risk; diabetes DSS^i^ pilot	8
Acceptability or attitudes only	Pre-post clinician experience; single-lead ECG^j^ survey	2
Study protocol	e-predictD	1
Conference abstract only	C the Signs; Thompson skin-lesion triage	2
Uncontrolled report, no comparator	Cruz 2019	1
Registration without published evaluation	NCT06828692	1
Not a learned or adaptive AI model	Neonatal bilirubin nomogram app; Caverly 2024 prediction-augmented SDM^k^	2
Concept or teaching paper	SMART-on-FHIR^l^ wearable-data visualization	1

^a^The complete itemized exclusion list is provided in Multimedia Appendix 1 [4-13].

^b^ML-CDSS: machine learning–based clinical decision support system.

^c^CDSS: clinical decision support system.

^d^HCV: hepatitis C virus.

^e^LLM: large language model.

^f^RCT: randomized controlled trial.

^g^PrEP: pre-exposure prophylaxis.

^h^CVD: cardiovascular disease.

^i^DSS: decision support system.

^j^ECG: electrocardiogram.

^k^SDM: shared decision-making.

^l^FHIR: Fast Healthcare Interoperability Resources.

### Characteristics of Included Studies

The AI-enabled evidence base comprised 10 studies spanning cardiovascular case-finding, dementia detection, melanoma and glaucoma screening, diabetic retinopathy screening, urinary tract infection management, HIV prevention, medication-related fall prevention, childhood asthma, and musculoskeletal pain. The systems included convolutional neural networks, deep computer vision models, natural language processing systems, electronic health record (EHR) risk models, interpretable machine learning classifiers, a case-based reasoning system, and one multicomponent CDSS containing a data-derived prediction model alongside guideline rules. Designs included 5 cluster-randomized trials, 1 individually randomized trial, 2 nonrandomized intervention evaluations, and 2 prospective diagnostic accuracy studies (Table 1).

### Risk of Bias

Risk-of-bias judgments are shown in Figure 2 and Multimedia Appendix 1 [4-13]. All 6 randomized trials were judged as having some concerns overall; for the 5 cluster trials, the additional identification or recruitment domain did not change the overall category. Herter et al [9] and Pinto et al [7] were at serious risk under ROBINS-I, driven principally by confounding and temporal change in their nonrandomized comparisons. Under QUADAS-2, Papachristou et al [6] was at high overall risk because of clinician-selected sampling and differential reference verification, with high applicability concern for its restricted lesion spectrum. Jan et al [8] was at high overall risk because only 277 of 414 participants contributed analyzable images; applicability was of low concern for the intended opportunistic primary care screening setting. Judgments from the 3 appraisal tools were kept separate.

**Figure 2:**
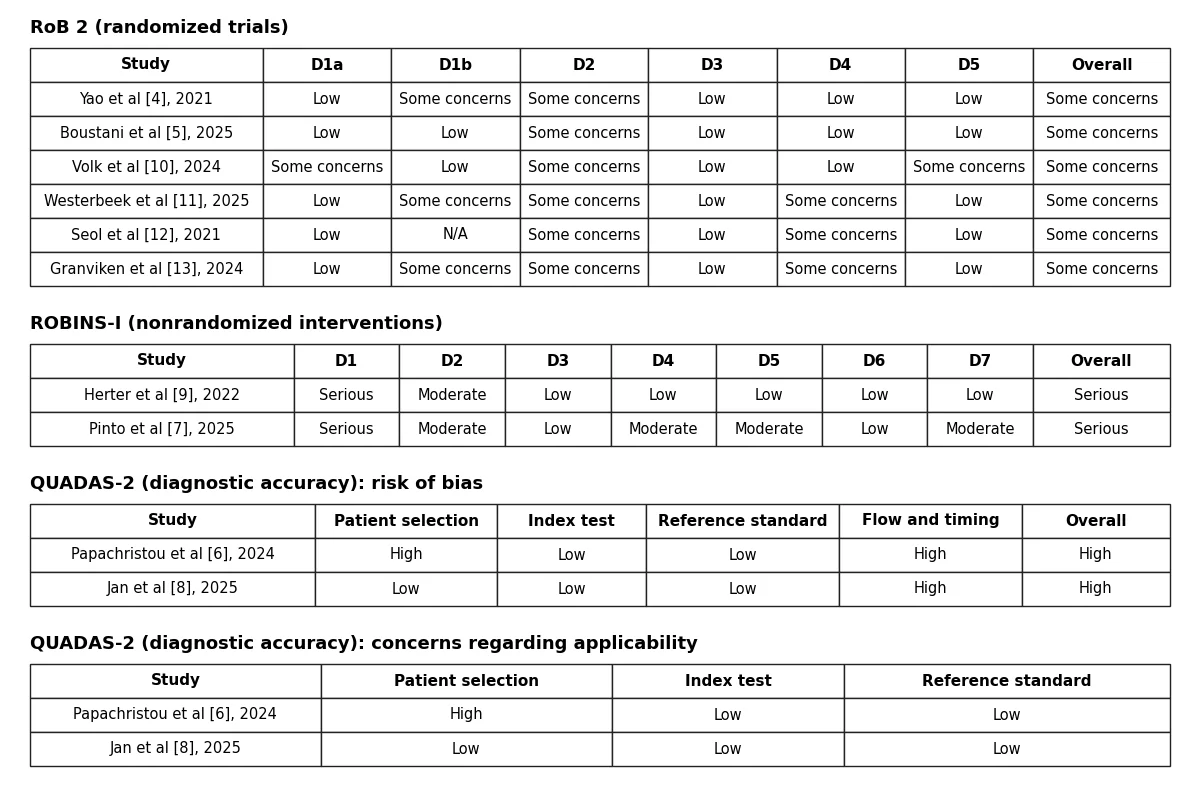
Risk of bias of the 10 AI-enabled studies [4-13] at domain level, separated by appraisal tool. Five cluster-randomized trials were assessed with the risk of bias 2 (RoB 2) cluster-randomized extension, including the participant identification and recruitment domain; Seol et al [12] used standard RoB 2. QUADAS-2 (Quality Assessment of Diagnostic Accuracy Studies 2) risk-of-bias and applicability judgments are displayed separately. The tools are not directly comparable. Full domain-level reasons are given in Multimedia Appendix 1. N/A: not applicable; ROBINS-I: Risk of Bias in Non-Randomized Studies of Interventions.

### Synthesis by Outcome

Case-finding results differed between the 2 randomized trials. AI electrocardiography increased new diagnoses of low ejection fraction (2.1% vs 1.6%; odds ratio [OR] 1.32, 95% CI 1.01-1.61; *P*=.007), with a stronger effect among patients flagged as high risk (OR 1.43, 95% CI 1.08-1.91) [4]. In contrast, an EHR machine learning passive digital marker for dementia did not increase new diagnosis when used alone (10.3% vs 12.4%; adjusted OR 0.84, 95% CI 0.63-1.11) and did not increase diagnostic assessment (27.8% vs 29%; adjusted OR 0.94, 95% CI 0.72-1.22). The combined arm, in which the marker was paired with a patient-reported instrument, increased new diagnosis to 15.4% (adjusted OR 1.31, 95% CI 1.05-1.64) [5].

Two studies assessed prospective diagnostic accuracy. A deep learning dermoscopy application used prospectively in Swedish primary care reached an area under the receiver operating characteristic curve (AUROC) of 0.960 for melanoma, but all lesions proceeded through standard work-up and the evaluation did not isolate a management effect [6]. In Australian general practice, the glaucoma system achieved an AUROC of 0.80, sensitivity of 65%, and specificity of 94.6% among 277 participants with analyzable images; the report went into the GP consultation, but image acquisition failed for a substantial proportion of recruited participants [8]. Neither study measured patient-important benefit attributable to the AI output.

At the clinician decision and care-process level, results were mixed and often difficult to attribute to the learned component alone. In a controlled before-after study, the interpretable urinary tract infection system was associated with increased treatment success from 75% to 80%, with a larger change in confirmed users (75% to 83%; *P*<.001) [9]. In the pre-exposure prophylaxis (PrEP) cluster trial, EHR machine learning prompts did not significantly increase initiation of PrEP care overall (6% vs 4.5%; hazard ratio 1.32, 95% CI 0.84-2.10), although a prespecified interaction favored clinicians already caring for people with HIV [10]. In the diabetic retinopathy program, NaIA-RD was deployed before GPs reviewed images and influenced the screening criteria of 3 of 4 GPs; agreement with GPs was at least 94.6% for nonreferral proposals but more variable for referral proposals. Because the study compared long periods before and after implementation without a concurrent control, the change could not be attributed to AI alone [7]. In the SNOWDROP cluster RCT, the multicomponent intervention improved shared decision-making scores for both GPs and patients (*P*<.001), reduced decisional conflict (*P*<.001), and improved communication satisfaction, but the primary medication-change analysis was inconclusive (OR 2.09, 95% CI 0.42-10.40) [11].

In a survey nested within the asthma trial, 28 of 42 clinicians responded and reported median estimated record review times of 3.5 minutes with the CDSS and 11.3 minutes without it (*P*<.001) [12]. This was a comparison of clinician estimates, including estimated time needed without the report, rather than objectively timed work or a randomized comparison of review time. Childhood asthma exacerbations did not differ between supported and usual care (12% vs 15%; OR 0.82, 95% CI 0.37-1.96) [12]. In SupportPrim, global perceived effect at 12 weeks was also not improved (55.4% vs 54.8%; adjusted OR 1.18, 95% CI 0.50-2.78). A clinically important improvement on the patient-specific functional scale occurred less often in the intervention arm (59.7% vs 70.3%; adjusted OR 0.41, 95% CI 0.20-0.85), favoring usual care, although the between-group difference was smaller than the trial’s prespecified 15% threshold for clinical importance [13]. Neither trial demonstrated patient-important benefit from AI-CDSS.

The 14 contextual deterministic studies covered cardiovascular risk and stroke or anticoagulation decisions [19,22,25,32], diabetes and hypertension [20,26], test ordering and kidney failure risk [21,23], liver disease [24,30], cervical screening follow-up [27], opioid use disorder care [28], ambulatory differential diagnosis [29], and general primary care diagnostic and treatment support [31].

### Implementation and Potential Harms

Implementation was incompletely measured across the AI-enabled evidence. Herter et al [9] reported a stronger effect among confirmed users; the SupportPrim trial and process evaluation by Granviken et al found low fidelity and use of the tool mainly to reinforce existing practice [13,33]; and Pinto et al [7] and Westerbeek et al [11] evaluated systems embedded in wider clinical workflows rather than isolated algorithmic components. In the study by Jan et al [8], only 66.9% of recruited participants yielded analyzable retinal images. Harms such as overdiagnosis, false positives, automation bias, inappropriate reassurance, and alert burden were rarely prespecified or quantified. Limited reporting prevented conclusions about safety. Per-study implementation observations are reported in Multimedia Appendix 1 [4-13].

### Certainty of Evidence

Certainty was low for diagnostic case-finding, diagnostic accuracy and screening, and patient-important clinical outcomes, and very low for clinician efficiency (Table 3 and Figure 3) [4-6,8,12,13]. The care-process category was not assigned a common GRADE rating because it combined treatment success, PrEP initiation, screening and referral decisions, and shared decision-making or medication change.

**Table 3:** Summary of findings: GRADE (Grading of Recommendations Assessment, Development and Evaluation) certainty for AI-enabled outcome bodies.

Outcome body	Studies	Participants	Effect	Certainty	GRADE rationale
Diagnostic case finding	Yao et al [4], 2021 and Boustani et al [5], 2025	22,641 and 5325	Low-EF^a^ diagnosis OR^b^ 1.32 (1.01-1.61); dementia algorithm-only arm OR 0.84 (0.63-1.11)	Low	Randomized evidence starts high; −1 for inconsistency (new diagnosis increased in 1 trial but not with the dementia algorithm alone); −1 for indirectness (detection without demonstrated patient benefit). Rating refers to whether AI-enabled case-finding increases new diagnoses of the target condition; the 2 trials disagreed, so the direction of effect is uncertain. No additional risk-of-bias downgrade: record-based outcome measurement was low risk in both trials; open-label delivery and Yao’s postallocation identification concerns were judged insufficient for a full-level downgrade.
Diagnostic accuracy and screening	Papachristou et al [6], 2024 and Jan et al [8], 2025	253 lesions and 277 analyzable participants	Melanoma AUROC^c^ 0.960; glaucoma AUROC 0.80, sensitivity 65%, and specificity 94.6%	Low	Start high; −1 for risk of bias (high overall QUADAS-2^d^ risk in both studies: clinician-selected sampling and differential verification in Papachristou; substantial flow and image-acquisition exclusions in Jan); −1 for indirectness (diagnostic discrimination and referral support do not themselves establish patient benefit). Rating refers to the conclusion that prospectively deployed classifiers can discriminate target conditions, but this evidence alone does not establish clinical benefit.
Clinician efficiency	Seol et al [12], 2021	28 of 42 clinicians responded; parent trial enrolled 184 children	Clinician-estimated review time: 3.5 vs 11.3 min (*P*<.001)	Very low	Survey comparison starts low: clinicians estimated review time with and without the tool; the comparison was not randomized. Downgraded for risk of bias (unblinded self-report and 28 of 42 responses) and indirectness (estimates of workload rather than observed review time), giving very low certainty. Rating refers to whether the asthma CDSS^e^ reduced clinicians’ record review time; the evidence is limited to clinician estimates from 1 trial.
Patient-important clinical outcomes	Seol et al [12], 2021 and Granviken et al [13], 2024	184 children and 724 adults	Asthma exacerbation OR 0.82 (0.37-1.96); GPE^f^ OR 1.18 (0.50-2.78); and functional improvement OR 0.41 (0.20-0.85), favoring usual care	Low	Randomized evidence starts high; −1 for risk of bias (some concerns, including unblinded clinicians, low fidelity, and patient-reported outcomes); −1 for imprecision. Rating refers to the conclusion that AI-enabled CDSS may make little or no difference to patient-important outcomes in these 2 settings; one Granviken functional end point favored usual care.

^a^EF: ejection fraction.

^b^OR: odds ratio.

^c^AUROC: area under the receiver operating characteristic curve.

^d^QUADAS-2: Quality Assessment of Diagnostic Accuracy Studies 2.

^e^CDSS: clinical decision support system.

^f^GPE: global perceived effect.

**Figure 3:**
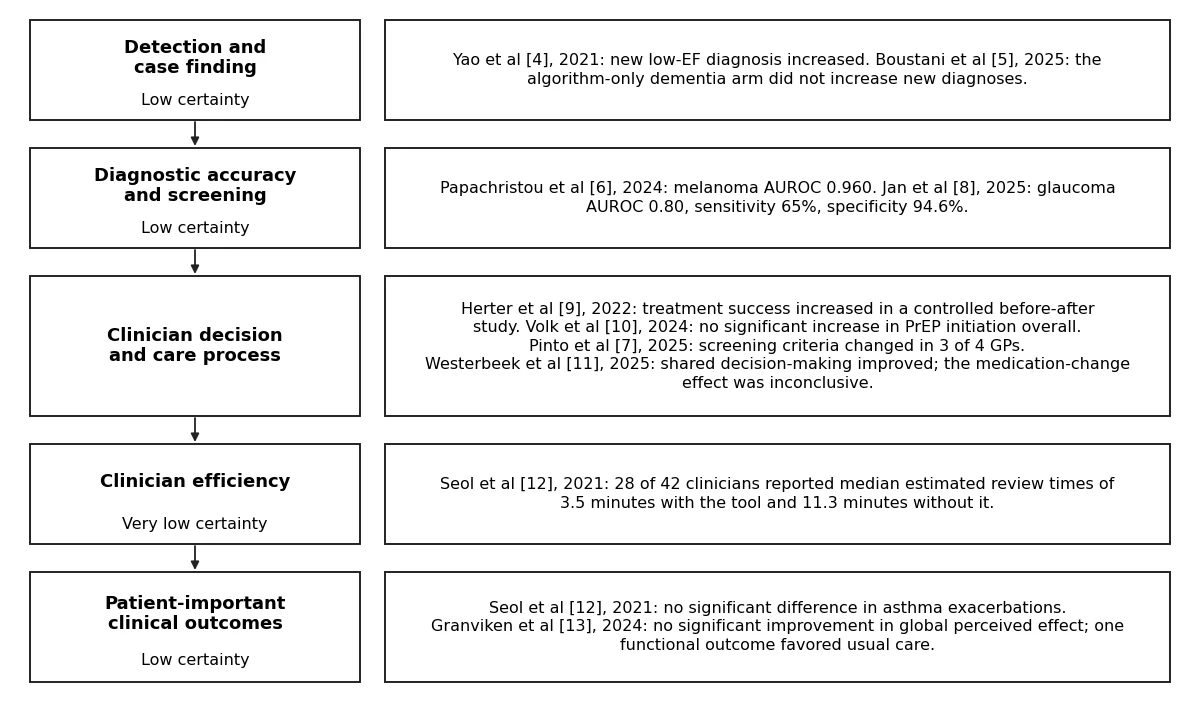
Evidence map of the 10 AI-enabled studies [4-13] across outcome levels. Certainty ratings correspond to Table 3. Care-process findings are synthesized descriptively because the end points differ. The vertical order organizes the outcome levels and does not imply a causal progression. AUROC: area under the receiver operating characteristic curve; EF: ejection fraction; GP: general practitioner; PrEP: pre-exposure prophylaxis.

## Discussion

### Principal Findings

The 10 AI-enabled studies reported mixed effects across outcome levels. Case-finding improved in the electrocardiogram trial but not with the dementia algorithm alone. Diagnostic discrimination varied by application, and care-process findings depended on the intervention and end point. Neither trial assessing patient-important outcomes demonstrated benefit. These results did not establish a consistent progression from diagnostic performance to improved patient outcomes.

Incomplete uptake, low fidelity, and multicomponent interventions limited interpretation of the observed effects. The studies did not distinguish these factors from model calibration, population differences, task selection, or intervention design. The evidence therefore could not identify a common cause for the differences between diagnostic, process, and patient outcomes.

### Interpretation and Implications

Figure 4 separates model output, clinician-facing support, changes in care, and patient-important outcomes. The framework identifies measurements needed to connect these stages. The included studies generally assessed different interventions and populations at different stages, so comparisons across outcome levels could not establish a causal pathway.

**Figure 4:**
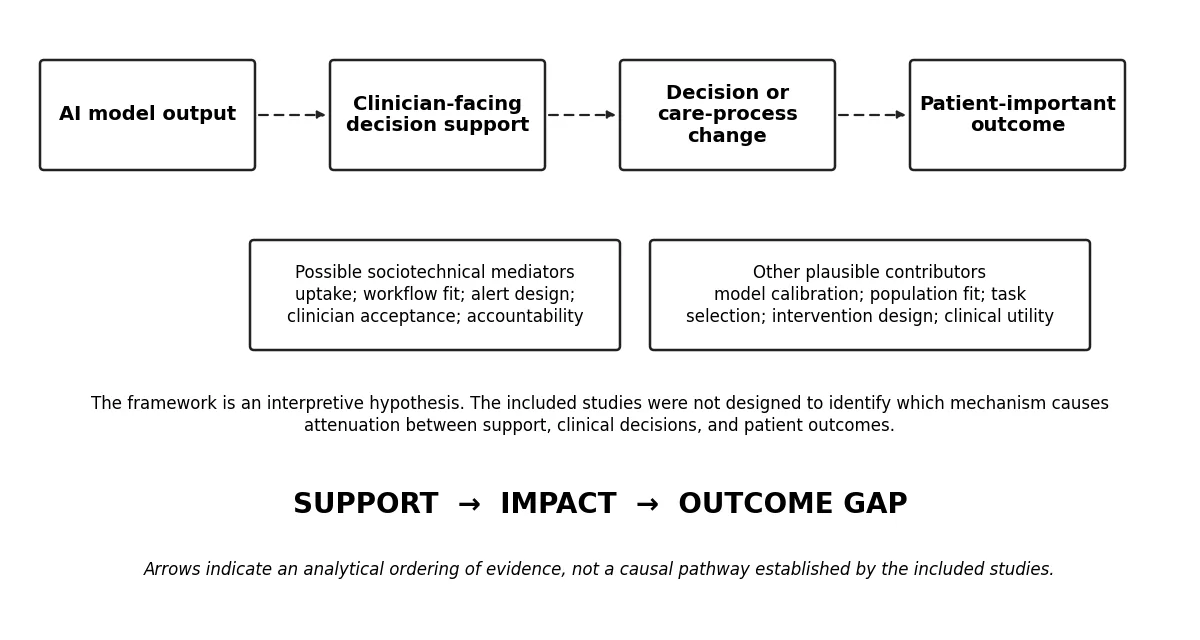
Proposed support-impact-outcome framework derived from the synthesis. Dashed arrows represent transitions that require empirical demonstration. Model, population, task, intervention, and sociotechnical factors are shown as possible contributors to attenuation rather than as mechanisms established by the included studies.

Low adherence to recommendations also requires interpretation. Clinicians may modify or reject advice because of patient preferences, comorbidity, competing risks, or information unavailable to the model. Evaluations should record these reasons and assess the appropriateness of decisions, alongside uptake and patient outcomes.

### Comparison With Prior Reviews

The mixed effects observed here are consistent with the broader findings of Susanto et al [1] and the heterogeneity of primary care implementations described by Gomez-Cabello et al [2]. This review separates learned and deterministic systems and evaluates certainty according to the outcome measured. It also identifies limited direct evidence connecting changes in care to patient-important benefit.

### Limitations

The small evidence base spans different conditions, interventions, designs, outcome definitions, and follow-up periods. These differences limit comparisons and certainty judgments across studies. Embase was not searched. The contextual deterministic studies were not formally appraised. Publication bias could not be reliably assessed in the small outcome bodies. The efficiency finding relied on an unblinded clinician survey with incomplete response.

### Conclusions

Evidence published through December 2025 did not establish consistent clinical benefit from AI-enabled CDSS in primary care. Some evaluations reported improvements in case-finding or care processes, but effects varied and patient-important benefit was not demonstrated. Future trials should measure changes in clinical decisions and patient outcomes within the same evaluation, with adequate follow-up and explicit assessment of uptake and harms.

## Appendix

Multimedia Appendix 1 Search strategies, study-level eligibility and excluded studies, PRISMA 2020 checklist, risk-of-bias domain-level assessments, and implementation characteristics and potential harms.

## Abbreviations

AUROC: area under the receiver operating characteristic curve
CDSS: clinical decision support system
GP: general practitioner
GRADE: Grading of Recommendations Assessment, Development and Evaluation
ICTRP: International Clinical Trials Registry Platform
OR: odds ratio
PrEP: pre-exposure prophylaxis
PRISMA: Preferred Reporting Items for Systematic Reviews and Meta-Analyses
QUADAS-2: Quality Assessment of Diagnostic Accuracy Studies 2
RCT: randomized controlled trial
RoB 2: risk of bias 2
ROBINS-I: Risk of Bias in Non-Randomized Studies of Interventions
WHO: World Health Organization

## Data Availability

The study-level findings used in this review are presented in the manuscript tables and Multimedia Appendix; original reports are identified in the references. The executed search strategies, full-text exclusions, and appraisal judgments are provided in the appendix. No individual participant data were collected.
