# Supplementary material for: Real-World Effectiveness of AI-Enabled Clinical Decision Support in Primary Care: Systematic Review
Source: J Med Internet Res. 2026 Oct 8;28:e98630. doi: 10.2196/98630 (PMC13691469; doi:10.2196/98630)
Supplement: Supplementary file 1 [file jmir_v28i1e98630_app1.docx]

**Appendix**

# **Section 1. Search strategies**

The final bibliographic and registry searches were executed on 18 August 2026, except CINAHL Ultimate, which was searched on 20 August 2026. The registered PROSPERO protocol specifies a publication window of 1 January 2011 through 31 December 2025. The upper limit defines a fixed, complete calendar-year publication window; the later searches allowed retrieval of records indexed after that boundary. No language limit was applied at the search stage. The registered protocol specifies no language restrictions on eligibility, and no full-text report was excluded on language grounds. Exact executed strings are reproduced below. Terms within concept blocks were combined with OR and the artificial-intelligence, clinical-decision-support, and primary/ambulatory-care blocks were combined with AND, except for the deliberately broader registry searches described in Section 1.7.

The iterative review by Xiong Xiaoyu, Subject Librarian, Tsinghua University Library, changed the search in several concrete ways: EBSCOhost was reclassified as a supplementary platform search rather than a database; terms were evaluated by database rather than assumed transferable; Web of Science was pruned where testing showed no contribution; Cochrane phrase-plus-truncation syntax was rebuilt with NEXT operators; the broader registry logic was made explicit; and CINAHL Ultimate was added to the final search set. Embase was not included in the formal review search set.

## **1.1 PubMed (NLM interface), 821 records; final run 18 August 2026**

(("Artificial Intelligence"[Mesh] OR "artificial intelligence"[tiab] OR "machine learning"[tiab] OR "deep learning"[tiab] OR "natural language processing"[tiab] OR "large language model*"[tiab] OR "neural network*"[tiab] OR "computer vision"[tiab] OR "case-based reasoning"[tiab] OR "case based reasoning"[tiab] OR "supervised learning"[tiab] OR "unsupervised learning"[tiab] OR "predictive model*"[tiab] OR "prediction model*"[tiab] OR "predictive algorithm*"[tiab]) AND ("Decision Support Systems, Clinical"[Mesh] OR "Decision Making, Computer-Assisted"[Mesh] OR "Diagnosis, Computer-Assisted"[Mesh] OR "Therapy, Computer-Assisted"[Mesh] OR "Medical Order Entry Systems"[Mesh] OR "decision support"[tiab] OR "decision-support"[tiab] OR "CDSS"[tiab] OR "clinical decision support*"[tiab] OR "computerized decision support"[tiab] OR "computerised decision support"[tiab] OR "computer-aided diagnos*"[tiab] OR "computer aided diagnos*"[tiab] OR "diagnostic support"[tiab] OR "decision aid"[tiab] OR "decision aids"[tiab] OR "clinical alert*"[tiab] OR "electronic reminder*"[tiab] OR "triage tool*"[tiab] OR "risk score*"[tiab]) AND ("Primary Health Care"[Mesh] OR "General Practice"[Mesh] OR "Family Practice"[Mesh] OR "Physicians, Primary Care"[Mesh] OR "Physicians, Family"[Mesh] OR "General Practitioners"[Mesh] OR "Ambulatory Care"[Mesh] OR "Ambulatory Care Facilities"[Mesh] OR "Community Health Services"[Mesh] OR "primary care"[tiab] OR "primary health care"[tiab] OR "primary healthcare"[tiab] OR "general practice*"[tiab] OR "general practitioner*"[tiab] OR "family medicine"[tiab] OR "family practice*"[tiab] OR "family physician*"[tiab] OR "ambulatory care"[tiab] OR "outpatient clinic*"[tiab] OR "community health cent*"[tiab] OR "primary care physiotherap*"[tiab])) AND ("2011/01/01"[Date - Publication] : "2025/12/31"[Date - Publication])

## **1.2 Scopus (Elsevier), 927 records; final run 18 August 2026**

TITLE-ABS-KEY ( "artificial intelligence" OR "machine learning" OR "deep learning" OR "natural language processing" OR "large language model*" OR "neural network*" OR "computer vision" OR "case-based reasoning" OR "case based reasoning" OR "supervised learning" OR "unsupervised learning" OR "predictive model*" OR "prediction model*" OR "predictive algorithm*" ) AND TITLE-ABS-KEY ( "decision support" OR "decision-support" OR "CDSS" OR "clinical decision support*" OR "computerized decision support" OR "computerised decision support" OR "computer-aided diagnos*" OR "computer aided diagnos*" OR "diagnostic support" OR "decision aid*" OR "clinical alert*" OR "electronic reminder*" OR "triage tool*" OR "risk score*" ) AND TITLE-ABS-KEY ( "primary care" OR "primary health care" OR "primary healthcare" OR "general practice" OR "general practitioner*" OR "family medicine" OR "family practice" OR "family physician*" OR "ambulatory care" OR "outpatient clinic*" OR "community health cent*" OR "primary care physiotherap*" ) AND PUBYEAR > 2010 AND PUBYEAR < 2026

*In Scopus, straight double quotation marks denote loose/approximate phrase searching within TITLE-ABS-KEY fields; exact phrase searching requires braces. PUBYEAR is exclusive at both bounds, so >2010 and <2026 returns 2011-2025 inclusive.*

## **1.3 Web of Science Core Collection (Clarivate), 634 records; final run 18 August 2026**

TS=(("artificial intelligence" OR "machine learning" OR "deep learning" OR "natural language processing" OR "large language model*" OR "neural network*" OR "computer vision" OR "case-based reasoning" OR "case based reasoning" OR "supervised learning" OR "unsupervised learning" OR "predictive model*" OR "prediction model*" OR "predictive algorithm*") AND ("decision support" OR "clinical decision support*" OR "CDSS" OR "computerized decision support" OR "computerised decision support" OR "computer-aided diagnos*" OR "computer aided diagnos*" OR "diagnostic support" OR "decision aid*" OR "clinical alert*" OR "electronic reminder*" OR "triage tool*" OR "risk score*") AND ("primary care" OR "primary health care" OR "primary healthcare" OR "general practice" OR "general practitioner*" OR "family medicine" OR "family practice" OR "family physician*" OR "ambulatory care" OR "outpatient clinic*" OR "community health cent*")) AND PY=(2011-2025)

*TS= searches title, abstract, author keywords, and Keywords Plus. The term "primary care physiotherap*" was removed from Web of Science after librarian testing showed that its removal did not change the result count.*

## **1.4 Supplementary EBSCOhost platform search (10 constituent databases), 203 records; 183 exportable; final run 18 August 2026**

((MH "Artificial Intelligence+") OR (MH "Machine Learning+") OR (MH "Neural Networks (Computer)+") OR (MH "Natural Language Processing+") OR TI ( "artificial intelligence" OR "machine learning" OR "deep learning" OR "natural language processing" OR "large language model*" OR "neural network*" OR "computer vision" OR "case-based reasoning" OR "case based reasoning" OR "predictive model*" OR "prediction model*" ) OR AB ( "artificial intelligence" OR "machine learning" OR "deep learning" OR "natural language processing" OR "large language model*" OR "neural network*" OR "computer vision" OR "case-based reasoning" OR "case based reasoning" OR "predictive model*" OR "prediction model*" )) AND ((MH "Decision Support Systems, Clinical+") OR (MH "Decision Making, Computer Assisted+") OR (MH "Diagnosis, Computer Assisted+") OR TI ( "decision support" OR "clinical decision support*" OR "CDSS" OR "computerized decision support" OR "computerised decision support" OR "computer-aided diagnos*" OR "diagnostic support" OR "decision aid*" OR "clinical alert*" OR "electronic reminder*" OR "risk score*" ) OR AB ( "decision support" OR "clinical decision support*" OR "CDSS" OR "computerized decision support" OR "computerised decision support" OR "computer-aided diagnos*" OR "diagnostic support" OR "decision aid*" OR "clinical alert*" OR "electronic reminder*" OR "risk score*" )) AND ((MH "Primary Health Care+") OR (MH "Family Practice+") OR (MH "Physicians, Family+") OR (MH "Ambulatory Care+") OR (MH "Community Health Services+") OR TI ( "primary care" OR "primary health care" OR "primary healthcare" OR "general practice*" OR "general practitioner*" OR "family medicine" OR "family practice*" OR "family physician*" OR "ambulatory care" OR "outpatient clinic*" OR "community health cent*" ) OR AB ( "primary care" OR "primary health care" OR "primary healthcare" OR "general practice*" OR "general practitioner*" OR "family medicine" OR "family practice*" OR "family physician*" OR "ambulatory care" OR "outpatient clinic*" OR "community health cent*" ))

Limiter: published date 20110101 to 20251231. Constituent databases and yields were Academic Search Ultimate (164), OpenDissertations (16), Library, Information Science and Technology Abstracts (8), Business Source Complete (4), OmniFile Full Text Select (4), Education Source (3), Communication and Mass Media Complete (1), ERIC (1), GreenFILE (1), and Regional Business News (1). Twenty of 203 records could not be exported in an available format and were screened manually in the platform; none met the eligibility criteria. EBSCOhost is therefore reported as a supplementary platform search, not a biomedical database. CINAHL Ultimate was searched separately on 20 August 2026 and is not included in this 203-record supplementary-platform total.

## **1.5 Cochrane Library, Search Manager, 111 CENTRAL trial records; final run 18 August 2026**

| **Line** | **Search** | **Yield** |
| --- | --- | --- |
| #1 | Artificial Intelligence [MeSH descriptor, exploded] | 5053 |
| #2 | ("artificial intelligence" OR "machine learning" OR "deep learning" OR "natural language processing" OR large NEXT language NEXT model* OR neural NEXT network* OR "case-based reasoning" OR predictive NEXT model*):ti,ab,kw | 12628 |
| #3 | #1 OR #2 | 13009 |
| #4 | Decision Support Systems, Clinical [MeSH descriptor, exploded] | 4029 |
| #5 | Decision Making, Computer-Assisted [MeSH descriptor, exploded] | 789 |
| #6 | ("decision support" OR "clinical decision support" OR CDSS OR "computerised decision support" OR "computerized decision support" OR "diagnostic support" OR decision NEXT aid* OR clinical NEXT alert*):ti,ab,kw | 6942 |
| #7 | #4 OR #5 OR #6 | 10035 |
| #8 | Primary Health Care [MeSH descriptor, exploded] | 97428 |
| #9 | General Practice [MeSH descriptor, exploded] | 30765 |
| #10 | ("primary care" OR "primary health care" OR general NEXT practice* OR general NEXT practitioner* OR "family medicine" OR family NEXT practice* OR family NEXT physician* OR "ambulatory care"):ti,ab,kw | 45330 |
| #11 | #8 OR #9 OR #10 | 129841 |
| #12 | #3 AND #7 AND #11; limited to Cochrane Library publication date Jan 2011-Dec 2025 and Trials (CENTRAL) | 111 |

Following the librarian comment on Cochrane Search Manager syntax, multiword expressions combining phrase searching and truncation were rewritten with NEXT operators. Reviews and Protocols are not included in the CENTRAL screening count and were used only for reference checking.

## **1.6 CINAHL Ultimate (EBSCO), 890 records; run 20 August 2026**

( (MH "Artificial Intelligence+") OR (MH "Machine Learning+") OR (MH "Neural Networks (Computer)+") OR (MH "Natural Language Processing+") OR TI ( "artificial intelligence" OR AI OR "machine learning" OR "deep learning" OR "natural language processing" OR "large language model*" OR "neural network*" OR "computer vision" OR "case-based reasoning" OR "case based reasoning" OR "supervised learning" OR "unsupervised learning" OR "predictive model*" OR "prediction model*" OR "predictive algorithm*" ) OR AB ( "artificial intelligence" OR AI OR "machine learning" OR "deep learning" OR "natural language processing" OR "large language model*" OR "neural network*" OR "computer vision" OR "case-based reasoning" OR "case based reasoning" OR "supervised learning" OR "unsupervised learning" OR "predictive model*" OR "prediction model*" OR "predictive algorithm*" ) ) AND ( (MH "Decision Support Systems, Clinical+") OR (MH "Decision Making, Computer Assisted+") OR (MH "Diagnosis, Computer Assisted+") OR TI ( "decision support" OR "decision-support" OR CDSS OR "clinical decision support*" OR "computerized decision support" OR "computerised decision support" OR "computer-aided diagnos*" OR "computer aided diagnos*" OR "diagnostic support" OR "decision aid*" OR "clinical alert*" OR "electronic reminder*" OR "triage tool*" OR "risk score*" ) OR AB ( "decision support" OR "decision-support" OR CDSS OR "clinical decision support*" OR "computerized decision support" OR "computerised decision support" OR "computer-aided diagnos*" OR "computer aided diagnos*" OR "diagnostic support" OR "decision aid*" OR "clinical alert*" OR "electronic reminder*" OR "triage tool*" OR "risk score*" ) ) AND ( (MH "Primary Health Care+") OR (MH "Family Practice+") OR (MH "Physicians, Family+") OR (MH "Ambulatory Care+") OR (MH "Community Health Services+") OR TI ( "primary care" OR "primary health care" OR "primary healthcare" OR "general practice*" OR "general practitioner*" OR "family medicine" OR "family practice*" OR "family physician*" OR "ambulatory care" OR "outpatient clinic*" OR "community health cent*" OR "primary care physiotherap*" ) OR AB ( "primary care" OR "primary health care" OR "primary healthcare" OR "general practice*" OR "general practitioner*" OR "family medicine" OR "family practice*" OR "family physician*" OR "ambulatory care" OR "outpatient clinic*" OR "community health cent*" OR "primary care physiotherap*" ) )

Interface: research.ebsco.com > CINAHL Ultimate > Basic Search. Limiter: published date 01/2011 to 12/2025. The 890 records were exported in two batches (500 and 390) with no duplicate accession numbers between batches.

## **1.7 Trial registries; final rerun 18 August 2026**

The trial registries were deliberately searched more broadly than the bibliographic databases because registry records contain less standardised indexing and often shorter descriptions. AI-related and clinical-decision-support terms were therefore combined in one block and ANDed with the primary-care block.

ClinicalTrials.gov Expert Search: ("artificial intelligence" OR "machine learning" OR "clinical decision support") AND ("primary care" OR "general practice") The query returned 437 records before the review date restriction; after the 2011-2025 restriction was applied manually, 433 records remained.

WHO ICTRP Search Portal (trialsearch.who.int): ("artificial intelligence" OR "machine learning" OR "clinical decision support") AND ("primary care" OR "general practice") The query returned 107 records representing 106 trials before the review date restriction; after the 2011-2025 restriction was applied manually, 104 trial records remained.

## **1.8 Search sensitivity and residual limitations**

Known eligible studies were used as a sensitivity check while the strategies were rebuilt. The corrected CENTRAL strategy returned 111 records after the date and Trials restrictions. Compared with the earlier 103-record CENTRAL run, this produced eight additional records. On 21 August 2026, the authors reconciled those eight records against the consolidated reference library and confirmed that all eight were duplicates of records already present and screened; the unique database/platform screening count therefore remained unchanged. One residual limitation is reported explicitly: the SupportPrim trial is indexed in CINAHL and can be found there by exact-title search but was not retrieved by the final three-concept CINAHL strategy. This indicates that the search is not exhaustive despite the expansion and librarian review. CINAHL nevertheless added three included evaluations to the final evidence set after full-text adjudication (Pinto 2025, Jan 2025, and Westerbeek 2025).

## 1.9 Source not included in the formal review search

Embase was not searched and is reported as a limitation in the manuscript.

## 1.10 Registration and subsequent methods

The review was registered in PROSPERO (CRD420261302104) on 9 February 2026, after the recorded review start of 6 November 2025. The registered record documents the publication window before the final August 2026 searches; registration should not be interpreted as prospective to review inception. PROSPERO version 1.1 was published on 31 July 2026. It records expansion beyond the original PubMed and Scopus search. The final methods additionally describe separate CINAHL searching, independent dual screening rather than the registered single-screen-plus-check procedure, and a separate deterministic contextual stream. These are reported as the final review methods rather than represented as unchanged prospective specifications. No language restriction was specified in the registered record.

# **Section 2. Study-level eligibility and excluded studies**

Table 2.1. Eligibility of the ten AI-enabled studies against the eligibility criteria

| **Study** | **AI component** | **Real-world design** | **Clinician-facing** | **Primary-care setting** | **Evaluable outcome** |
| --- | --- | --- | --- | --- | --- |
| Yao 2021 [4] | Deep CNN applied to ECG | Cluster RCT | Yes | Low ejection fraction; primary care | New low-EF diagnosis 2.1% vs 1.6%; OR 1.32 (95% CI 1.01-1.61). |
| Boustani 2025 [5] | EHR machine-learning passive digital marker | Three-arm cluster RCT | Yes | Dementia detection; FQHC primary care | Algorithm-only arm: new diagnosis 10.3% vs 12.4%; adjusted OR 0.84 (0.63-1.11). Combined marker + patient instrument arm: 15.4%; OR 1.31 (1.05-1.64). |
| Papachristou 2024 [6] | Deep learning / computer vision | Prospective diagnostic-accuracy study | Yes | Melanoma; 36 primary care centres | AUROC 0.960; all lesions underwent standard work-up, so management effect was not isolated. |
| Pinto 2025 [7] | Deep-learning diabetic-retinopathy screening system (NaIA-RD) | Uncontrolled (historical) before-after study | Yes: AI referral proposal shown to GP before image review | Diabetic retinopathy screening; primary care | AI influenced screening criteria in 3 of 4 GPs; non-referral agreement >=94.6%; historical before-after design precludes causal attribution to AI alone. |
| Jan 2025 [8] | Deep-learning fundus-image glaucoma classifier | Prospective pragmatic diagnostic-accuracy study | Yes: report accompanied patient into GP consultation / physician portal access | Glaucoma screening; two general-practice clinics | AUROC 0.80; sensitivity 65.0%; specificity 94.6%. AI report accompanied the patient into the GP consultation; 66.9% had analysable images. |
| Herter 2022 [9] | Interpretable ML decision-tree classifiers | Controlled before-after study | Yes | Urinary tract infection; primary care | Treatment success increased from 75% to 80%; confirmed-use subgroup 75% to 83% (P<.001). |
| Volk 2024 [10] | EHR machine-learning HIV risk-prediction model | Cluster RCT | Yes | HIV PrEP; primary care | PrEP-care initiation 6.0% vs 4.5%; HR 1.32 (0.84-2.10); prespecified interaction by prior HIV-care experience. |
| Westerbeek 2025 [11] | EHR CDSS with integrated data-derived fall-risk prediction model plus guideline rules | Cluster RCT | Yes: EHR-integrated CDSS used by GP in consultation | Medication-related fall prevention; general practice | Higher GP and patient shared-decision-making scores (both P<.001) and lower decisional conflict (P<.001); medication-change effect inconclusive, OR 2.09 (0.42-10.40). |
| Seol 2021 [12] | NLP + machine learning | Individual RCT | Yes | Childhood asthma; primary care | Clinician-estimated review time 3.5 vs 11.3 min (P<.001); exacerbations 12% vs 15%, OR 0.82 (0.37-1.96). |
| Granviken 2024 [13] | Case-based reasoning from a library of prior cases | Cluster RCT | Yes: physiotherapist used web CDSS in consultation | Musculoskeletal pain; primary care physiotherapy | GPE: adjusted OR 1.18 (0.50-2.78), null. Clinically important functional improvement: OR 0.41 (0.20-0.85), favouring usual care; between-group difference below prespecified 15% threshold. |

## **Adjudicated borderline records**

| **Record** | **Disposition** | **Reason** |
| --- | --- | --- |
| Megerian 2022 | Exclude | Participating HCPs supplied an input but were blinded to the device output during the diagnostic validation; output did not function as clinician-facing support used in care. |
| Pinto 2025 | Include | NaIA-RD was deployed in the routine diabetic-retinopathy screening workflow; GPs saw the AI screening proposal before assessing images and the evaluation measured real screening decisions. |
| Jan 2025 | Include | AI report was taken into the GP consultation and was also physician-accessible online; the study prospectively evaluated accuracy and referral support in general practice. |
| Westerbeek 2025 | Include, multicomponent | General-practice cluster RCT of EHR CDSS containing an integrated data-derived fall-risk prediction model plus guideline rules and a patient portal. Effects cannot be attributed to the learned component alone. |
| Caverly 2024 | Exclude from AI population | Prediction-augmented decision-support tool relied on a conventional fixed prediction model and benefit categories; it did not meet the review definition of learned/adaptive AI. |

**Table 2.2. Full-text reports excluded after adjudication (n=42)**

| **#** | **Study / identifier** | **Reason** |
| --- | --- | --- |
| 1 | Susanto 2025 (10.2196/73131) | Simulation. Nine clinical vignettes using a conceptual prototype; no patient managed. |
| 2 | Sore-throat ML-CDSS (10.1186/s12879-025-12335-x) | Simulation. 211 physicians responded to four hypothetical scenarios with a simulated CDSS output. |
| 3 | AI fundus screening (10.3390/bioengineering12121304) | Reader study. Four clinicians read 500 de-identified fundus photographs twice, unaided then AI-assisted. |
| 4 | SPIRO-AID (10.1056/AIoa2400804) | Simulation. 234 primary care clinicians interpreted de-identified spirometry records on an online platform; no real consultation was altered. |
| 5 | Low-back-pain self-referral models (10.1016/j.ijmedinf.2017.11.010) | Simulation. Machine-learning models compared on fictive cases collected in a vignette study. |
| 6 | Simao 2025 (10.1186/s12875-025-02963-2) | Benchmark. Cross-sectional comparison of ChatGPT output with physician decisions; the model informed no consultation. |
| 7 | DermaAId genital dermatoses (10.1111/ijd.17060) | Benchmark. App diagnoses compared with primary care physicians and dermatologists against a consensus standard. |
| 8 | Escale-Besa 2023 (10.1038/s41598-023-31340-1) | Benchmark. GPs recorded a diagnosis before the image was uploaded; AI outputs were compared afterwards. |
| 9 | Sunjaya 2025 (10.1183/23120541.00116-2025) | Validation without prospective clinical use. Retrospective blinded diagnostic validation against pulmonologist consensus. |
| 10 | POLAR Diversion (10.1055/s-0039-1678608) | Development and internal validation of a machine-learning risk tool without prospective clinical use. |
| 11 | selfBACK (10.1001/jamainternmed.2021.4097) | Not clinician-facing. Randomised trial of a patient-facing self-management app. |
| 12 | Telehealth matched cohort (10.1136/bmjopen-2015-009221) | Not a CDSS. Machine learning was used for analytic control matching, not decision support in the consultation. |
| 13 | Wang 2019 (10.1136/bmjqs-2019-009367) | Not clinician-facing in primary care. Machine-learning case-finding output was delivered to a hospital anticoagulation-service pharmacist. |
| 14 | Zaman 2025 (10.1186/s12889-025-22770-9) | Not clinician-facing. Community health worker intervention. |
| 15 | Gerdeskold 2020 (10.1186/s12911-020-01313-9) | Not a clinician-facing decision support system under the prespecified definition. |
| 16 | Megerian 2022 (10.1038/s41746-022-00598-6) | Prospective diagnostic validation in a primary-care population, but participating clinicians were blinded to the device output; the AI therefore did not function as clinician-facing decision support used in care during the evaluation. |
| 17 | Epilepsy surgery alerts (10.1111/epi.17629) | Specialist setting: paediatric neurology outpatient clinics. |
| 18 | Smart Scope colposcopy (10.7759/cureus.81212) | Hospital obstetrics and gynaecology setting; also a single-arm pilot. |
| 19 | Benrimoh AID-ME (10.4088/JCP.24m15634) | Conducted, by the authors’ report, without primary care clinicians. |
| 20 | LLM diagnostic decision support RCT (10.3233/SHTI250844) | Emergency medicine setting, not primary care. |
| 21 | Myers 2026 (10.1016/j.cct.2025.108209) | Feasibility pragmatic trial; also published after the 31 December 2025 upper date limit and therefore outside the eligible publication window. |
| 22 | COVID home-isolation CDSS (10.3390/jcm13195974) | Feasibility evaluation; patient self-reporting rather than clinician-facing support. |
| 23 | Bilaver iREACH (10.1111/pai.14115) | Practice-based feasibility trial design. |
| 24 | Jaremko hip dysplasia (10.1038/s41598-023-35603-9) | Small pilot of beta software with no reference standard applied to the cohort and no comparator. |
| 25 | Krakower PrEP pilot (10.1056/CAT.23.0215) | Pilot feasibility and clinician-acceptance report; outcome analyses were stated as ongoing. |
| 26 | FIND-AF pilot (10.1136/openhrt-2023-002447) | Pilot, non-randomised, single-arm evaluation. |
| 27 | Retinal photography CVD risk (10.1038/s41746-025-01436-1) | Feasibility and acceptability; accuracy assessed against an external cohort rather than outcomes in the recruited sample. |
| 28 | Diabetes DSS pilot (10.1109/JTEHM.2020.3031107) | Pilot evaluation. |
| 29 | Pre-post provider experience (10.1016/j.ijmedinf.2019.104072) | Attitudes only; no clinical, behavioural, or process outcome. |
| 30 | Single-lead ECG survey (10.1016/j.acvd.2025.05.008) | Acceptability only; survey of perceived utility. |
| 31 | e-predictD (10.3389/fpsyt.2023.1163800) | Study protocol; no outcome data reported. |
| 32 | C the Signs (10.1200/JCO.2024.42.16_suppl.1560) | Conference abstract only; no full report retrieved. |
| 33 | Thompson skin lesion triage (iProceedings 2021) | Conference abstract only; two-page proceedings item. |
| 34 | Cruz 2019 (10.3233/SHTI190285) | Uncontrolled report; no comparator, so an effect estimate could not be made. |
| 35 | NCT06828692 antihypertensive CDSS | Trial registration without a published evaluation. |
| 36 | OpenEvidence platform evaluation (10.1177/21501319251333515) | Simulation. Five patient cases analysed retrospectively and rated by physicians; not deployment. |
| 37 | Medicaid impactability model (10.1089/pop.2017.0122) | Development and validation of a predictive model without prospective clinical use. |
| 38 | Hepatitis C identification programme, Cherokee Nation (MMWR 2016;65:461-466) | Not a clinician-facing decision support system; a screening and linkage-to-care programme. |
| 39 | Integrated personalised care model (Int J Integr Care 2022) | Not a clinician-facing decision support system; a service-model description. |
| 40 | EHR add-on app for neonatal bilirubin (10.1001/jamanetworkopen.2019.15343) | Not a learned model; the app implements a fixed nomogram. |
| 41 | Caverly 2024 (10.1001/jamanetworkopen.2024.19624) | Not AI under the operational definition. The deployed tool used a conventional fixed prediction model and benefit categories rather than a learned/adaptive AI component. |
| 42 | SMART-on-FHIR wearable-data visualisation (Stud Health Technol Inform 2022) | Concept and teaching paper; no clinical evaluation. |

# **Section 3. PRISMA 2020 checklist**

| **#** | **PRISMA item** | **Location** |
| --- | --- | --- |
| 1 | Title | Title identifies the report as a systematic review |
| 2 | Abstract | Structured abstract |
| 3 | Rationale | Introduction |
| 4 | Objectives | Introduction, final paragraph |
| 5 | Eligibility criteria | Methods, Inclusion criteria and Exclusion criteria |
| 6 | Information sources | Methods; Appendix Section 1 |
| 7 | Search strategy | Appendix Section 1 |
| 8 | Selection process | Methods, Study selection and data extraction |
| 9 | Data collection | Methods, Study selection and data extraction |
| 10 | Data items | Methods, extraction fields |
| 11 | Risk of bias | Methods; Figure 2; Appendix Section 4 |
| 12 | Effect measures | Results; Tables 1 and 3 |
| 13 | Synthesis methods | Methods; Results, Synthesis by outcome |
| 14 | Reporting bias assessment | Methods; Table 3 |
| 15 | Certainty assessment | Methods; Table 3 |
| 16 | Study selection | Results; Figure 1 |
| 17 | Study characteristics | Results; Table 1 |
| 18 | Risk of bias in studies | Results; Figure 2 |
| 19 | Results of individual studies | Results; Table 1 |
| 20 | Results of syntheses | Results; Table 3 |
| 21 | Reporting biases | Methods, Risk of bias and certainty of evidence; Discussion, Limitations. No formal publication-bias analysis. |
| 22 | Certainty of evidence | Results; Table 3 |
| 23 | Discussion | Discussion |
| 24 | Registration and protocol | Abstract; Methods; Appendix Section 1.10 (registration and subsequent methods) |
| 25 | Support | Declarations |
| 26 | Competing interests | Declarations |
| 27 | Availability of materials | Appendix Sections 1-5 |

# **Section 4. Risk-of-bias domain-level assessments**

The ten AI-enabled studies were assessed with design-matched instruments. Five of the six randomised studies were cluster-randomised and were assessed with the RoB 2 cluster-randomised extension, including the domain for bias arising from identification or recruitment of individual participants after cluster allocation. Seol 2021 was assessed with standard RoB 2. ROBINS-I is reported separately for non-randomised intervention studies. For QUADAS-2, risk-of-bias judgments and concerns regarding applicability are reported separately. These instruments do not share a common domain structure or a common meaning of category labels.

## **RoB 2: randomised trials**

| **Study** | **Domain** | **Judgment** | **Basis** |
| --- | --- | --- | --- |
| Yao et al 2021 [4] | D1a Randomisation process | Low | Primary-care teams were cluster-randomised. |
|  | D1b Identification/recruitment | Some concerns | Patients entered after cluster allocation when routine-care ECGs were obtained. There was no formal patient recruitment, but ECG acquisition was clinician-dependent and allocation was known. |
|  | D2 Deviations | Some concerns | Open-label decision support; clinician action discretionary. |
|  | D3 Missing data | Low | Outcome from routine records. |
|  | D4 Measurement | Low | Objective new diagnosis of low ejection fraction. |
|  | D5 Reporting | Low | Pragmatic trial with prespecified outcomes. |
|  | Overall | Some concerns | Driven by D1b and D2; the added cluster domain did not change the overall judgment. |
| Boustani et al 2025 [5] | D1a Randomisation process | Low | Nine clinics were assigned by a computer-generated randomization scheme. |
|  | D1b Identification/recruitment | Low | All eligible patients visiting the randomised clinics were included using prespecified EHR criteria under a waiver of consent; there was no discretionary patient recruitment. |
|  | D2 Deviations | Some concerns | Open-label clinic intervention; fidelity of marker review not fully reported. |
|  | D3 Missing data | Low | Clinic-level follow-up complete in reported analysis. |
|  | D4 Measurement | Low | EHR-coded diagnosis/assessment applied consistently. |
|  | D5 Reporting | Low | Primary and secondary outcomes reported across arms. |
|  | Overall | Some concerns | Driven by D2; the added cluster domain did not change the overall judgment. |
| Volk et al 2024 [10] | D1a Randomisation process | Some concerns | Provider-level cluster allocation; sequence generation and concealment were not described in the available report. |
|  | D1b Identification/recruitment | Low | Eligible appointments were identified by prespecified EHR risk and scheduling criteria after provider allocation, without active patient recruitment. |
|  | D2 Deviations | Some concerns | Providers unblinded to prompt; prompt-handling fidelity not reported. |
|  | D3 Missing data | Low | EHR outcome available for eligible appointments. |
|  | D4 Measurement | Low | Objective care-process outcome from records. |
|  | D5 Reporting | Some concerns | Prespecified interaction reported; protocol/analysis plan not available for comparison. |
|  | Overall | Some concerns | Driven by D1a, D2 and D5; the added cluster domain did not change the overall judgment. |
| Westerbeek et al 2025 [11] | D1a Randomisation process | Low | Six practices were randomised 1:1 using the reported randomization procedure; the small number of clusters alone was not treated as bias. |
|  | D1b Identification/recruitment | Some concerns | Practices knew allocation before inviting patients and initiated patient contact; the number approached was not tracked consistently in one practice. |
|  | D2 Deviations | Some concerns | Open-label, multicomponent intervention; advice-reading fidelity cannot isolate the prediction-model component. |
|  | D3 Missing data | Low | 84 of 90 baseline participants included in analysis. |
|  | D4 Measurement | Some concerns | Audio-coded shared decision-making had high interobserver agreement; some outcomes were patient-reported and the intervention was not blinded. |
|  | D5 Reporting | Low | Trial preregistered (NCT05611008) and main prespecified outcomes reported. |
|  | Overall | Some concerns | Driven by D1b, D2 and D4; the added cluster domain did not change the overall judgment. |
| Seol et al 2021 [12] | D1 Randomisation process | Low | Individual randomization reported; cluster-specific D1b is not applicable. |
|  | D2 Deviations | Some concerns | Open-label clinician-facing support. |
|  | D3 Missing data | Low | Complete follow-up reported. |
|  | D4 Measurement | Some concerns | Exacerbation ascertainment in open-label design. |
|  | D5 Reporting | Low | Primary outcome prespecified. |
|  | Overall | Some concerns | Driven by D2 and D4. |
| Granviken et al 2024 [13] | D1a Randomisation process | Low | Computer-generated cluster allocation, stratified by manual-therapy specialty; physiotherapists were recruited before allocation. |
|  | D1b Identification/recruitment | Some concerns | Patients were recruited after therapists knew allocation. Consecutive recruitment and patient blinding reduced, but did not remove, the potential for identification/recruitment bias. |
|  | D2 Deviations | Some concerns | Therapists unblinded; process evaluation found low fidelity and use to reinforce existing practice. |
|  | D3 Missing data | Low | 619/724 (85%) completed 12-week follow-up; intention-to-treat approach. |
|  | D4 Measurement | Some concerns | Primary outcomes patient-reported; therapists unblinded. |
|  | D5 Reporting | Low | Published statistical analysis plan; ISRCTN17927832. |
|  | Overall | Some concerns | Driven by D1b, D2 and D4; the added cluster domain did not change the overall judgment. |

## **ROBINS-I: non-randomised intervention studies**

| **Study** | **Domain** | **Judgment** | **Basis** |
| --- | --- | --- | --- |
| Herter et al 2022 [9] | D1 Confounding | Serious | Before-after exposure with secular trend; matched controls reduce but do not remove confounding. |
|  | D2 Selection | Moderate | Practice-level participation. |
|  | D3 Classification | Low | Clear intervention classification. |
|  | D4 Deviations | Low | Routine deployment. |
|  | D5 Missing data | Low | Outcome records available. |
|  | D6 Outcome measurement | Low | Treatment success from records. |
|  | D7 Reporting | Low | Primary reported effect consistent with design. |
|  | Overall | Serious | Driven by confounding/temporal trend. |
| Pinto et al 2025 [7] | D1 Confounding | Serious | Historical before-after comparison without concurrent control; secular changes, workflow evolution, and the pandemic period could affect screening. |
|  | D2 Selection | Moderate | Long-running regional screening programme with stable but selected GP screeners. |
|  | D3 Classification | Low | AI deployment date and workflow were clearly defined. |
|  | D4 Deviations | Moderate | Clinical workflow and experience with the system evolved after deployment. |
|  | D5 Missing data | Moderate | Availability of AI grades and data completeness differed across periods. |
|  | D6 Outcome measurement | Low | Referral/screening decisions recorded in the health information system. |
|  | D7 Reporting | Moderate | Multiple real-world performance and process analyses reported. |
|  | Overall | Serious | Confounding and temporal change prevent causal attribution to AI alone. |

## **QUADAS-2: diagnostic-accuracy studies**

| **Study** | **Domain** | **Judgment** | **Basis** |
| --- | --- | --- | --- |
| Papachristou et al 2024 [6] | Patient selection | High | PCPs enrolled lesions already raising melanoma suspicion; consecutive/random sampling was not reported and several lesion/patient categories were excluded. |
|  | Index test | Low | AI threshold was prespecified and the app was applied prospectively. |
|  | Reference standard | Low | Histopathology or specialist clinical diagnosis followed the routine diagnostic pathway. |
|  | Flow and timing | High | Reference verification was differential: 134 lesions were diagnosed histopathologically and 119 by dermatologist clinical diagnosis, so not all lesions received the same reference standard. |
|  | Overall risk of bias | High | Driven by clinician-selected sampling not reported as consecutive, and differential reference verification. |
| Jan et al 2025 [8] | Patient selection | Low | Adults aged 50 years or older attending two GP clinics were prospectively recruited; no case-control sampling was used. |
|  | Index test | Low | Prospectively specified AI risk classification; reference graders were masked to AI output. |
|  | Reference standard | Low | Two registered optometrists independently graded images, with ophthalmologist adjudication of disagreements. |
|  | Flow and timing | High | Only 277/414 (66.9%) participants contributed analysable images; excluded participants were older and differed in ocular history. |
|  | Overall risk of bias | High | Driven by substantial flow/image-acquisition exclusions. |

QUADAS-2: concerns regarding applicability

| Study | Domain | Concern | Basis |
| --- | --- | --- | --- |
| Papachristou et al 2024 [6] | Patient selection | High | The study was restricted to lesions already considered melanoma-suspicious and excluded Fitzpatrick skin types V-VI, limiting applicability to unselected primary-care lesion assessment. |
|  | Index test | Low | The app was used prospectively by primary-care physicians in the intended clinical setting. |
|  | Reference standard | Low | The routine histopathology/dermatologist diagnostic pathway is relevant to the review question. |
| Jan et al 2025 [8] | Patient selection | Low | Routine GP attendees aged 50 years or older matched the intended opportunistic primary-care screening setting. |
|  | Index test | Low | The automated camera and AI risk report were used as intended in primary care. |
|  | Reference standard | Low | Independent expert image grading addressed the review question of referable glaucoma screening. |

Outcome-specific note for Seol 2021: the RoB 2 rows above concern the randomised patient outcome. The efficiency estimate came from a clinician survey (28/42 responses), using unblinded estimates of review time with and without the report. It was not an objectively timed or randomised review-time comparison. These outcome-specific concerns are reflected in the Very low certainty rating in Table 3.

# **Section 5. Implementation characteristics and potential harms**

| **Study** | **Delivery / integration** | **Uptake or fidelity** | **Potential harms / limitations** |
| --- | --- | --- | --- |
| Yao et al 2021 [4] | AI-ECG result surfaced to care team in routine practice; action discretionary. | Larger diagnostic effect in AI-positive subgroup than overall. | Downstream testing increased as intended; false positives/overdiagnosis not prespecified. |
| Boustani et al 2025 [5] | Passive marker run on EHR data and surfaced to primary-care team. | Algorithm-only arm produced no detectable change in diagnosis or assessment. | Potential overdiagnosis not formally assessed. |
| Papachristou et al 2024 [6] | AI dermatoscopy used by primary-care physician during consultation. | All lesions underwent standard work-up; tool-driven management fidelity not isolated. | False reassurance not evaluable because management did not depend on the tool. |
| Pinto et al 2025 [7] | NaIA-RD referral proposal displayed to GP before image review; integrated in screening workflow. | Influenced screening criteria in 3/4 GPs; non-referral agreement >=94.6%. | Historical design cannot isolate AI from temporal changes; inappropriate referral/non-referral remains a potential harm. |
| Jan et al 2025 [8] | AI fundus report accompanied patient into GP consultation and was physician-accessible online. | Only 66.9% had analysable images; follow-up depended on physician discretion and specialist adherence. | False positives 5% at person level; five false negatives reported; image-acquisition failure could deny screening. |
| Herter et al 2022 [9] | ML UTI classifier embedded in practice workflow. | Effect larger in confirmed-use subgroup (75% to 83%) than overall (75% to 80%). | Antibiotic over/undertreatment plausible but not prespecified or quantified. |
| Volk et al 2024 [10] | EHR staff message sent to PCP before upcoming visit. | A prespecified interaction favoured providers already caring for people with HIV; variable engagement. | Alert burden not assessed. |
| Westerbeek et al 2025 [11] | EHR-integrated CDSS plus patient portal used in fall-risk medication consultation. | All intervention GPs used CDSS; multicomponent design prevents attribution to prediction model alone. | Medication changes were inconclusive; small cluster sample and complex advice may affect generalisability. |
| Seol et al 2021 [12] | NLP/ML tool surfaced guideline status at visit. | Lower clinician-estimated review burden; 28/42 clinicians responded to the survey, without objective timing. | No specific harms reported; exacerbation outcome null. |
| Granviken et al 2024 [13] | Standalone web CDSS opened by physiotherapist during consultation. | Low fidelity; process evaluation found use mainly to reinforce existing practice. | No adverse events collected; functional endpoint favoured usual care, so harm cannot be excluded. |

# **References**

4. Yao X, Rushlow DR, Inselman JW, McCoy RG, Thacher TD, Behnken EM, et al. Artificial intelligence-enabled electrocardiograms for identification of patients with low ejection fraction: a pragmatic, randomized clinical trial. Nat Med. May 2021;27(5):815-819. [doi: 10.1038/s41591-021-01335-4] [Medline: 33958795]

5. Boustani MA, Ben Miled Z, Owora AH, Fowler NR, Dexter P, Puster E, et al. Digital detection of dementia in primary care: a randomized clinical trial. JAMA Netw Open. Nov 03, 2025;8(11):e2542222. [doi: 10.1001/jamanetworkopen.2025.42222] [Medline: 41212562]

6. Papachristou P, Söderholm M, Pallon J, Taloyan M, Polesie S, Paoli J, et al. Evaluation of an artificial intelligence-based decision support for the detection of cutaneous melanoma in primary care: a prospective real-life clinical trial. Br J Dermatol. Jun 20, 2024;191(1):125-133. [doi: 10.1093/bjd/ljae021] [Medline: 38234043]

7. Pinto I, Olazarán Á, Jurío D, De la Osa B, Sainz M, Oscoz A, et al. Improving diabetic retinopathy screening using artificial intelligence: design, evaluation and before-and-after study of a custom development. Front Digit Health. Jun 19, 2025;7:1547045. [doi: 10.3389/fdgth.2025.1547045] [Medline: 40613078]

8. Jan CL, Joseph S, Vingrys AJ, Henwood J, Ge Z, Stafford RS, et al. Prospective pragmatic trial of automated retinal photography and AI glaucoma screening in Australian primary care. NPJ Digit Med. Jul 01, 2025;8(1):386. [doi: 10.1038/s41746-025-01768-y] [Medline: 40595445]

9. Herter WE, Khuc J, Cinà G, Knottnerus BJ, Numans ME, Wiewel MA, et al. Impact of a machine learning-based decision support system for urinary tract infections: prospective observational study in 36 primary care practices. JMIR Med Inform. May 04, 2022;10(5):e27795. [doi: 10.2196/27795] [Medline: 35507396]

10. Volk JE, Leyden WA, Lea AN, Lee C, Donnelly MC, Krakower DS, et al. Using electronic health records to improve HIV preexposure prophylaxis care: a randomized trial. J Acquir Immune Defic Syndr. Apr 01, 2024;95(4):362-369. [doi: 10.1097/QAI.0000000000003376] [Medline: 38412047]

11. Westerbeek L, Linn AJ, van Weert HC, van der Velde N, Medlock S, Abu-Hanna A, et al. A randomized controlled trial to evaluate innovative decision support in the context of fall prevention. NPJ Digit Med. Jul 11, 2025;8(1):431. [doi: 10.1038/s41746-025-01822-9] [Medline: 40646167]

12. Seol HY, Shrestha P, Muth JF, Wi CI, Sohn S, Ryu E, et al. Artificial intelligence-assisted clinical decision support for childhood asthma management: a randomized clinical trial. PLoS One. Aug 02, 2021;16(8):e0255261. [doi: 10.1371/journal.pone.0255261] [Medline: 34339438]

13. Granviken F, Meisingset I, Bach K, Bones AF, Simpson MR, Hill JC, et al. Personalised decision support in the management of patients with musculoskeletal pain in primary physiotherapy care: a cluster randomised controlled trial (the SupportPrim project). Pain. May 01, 2025;166(5):1167-1178. [doi: 10.1097/j.pain.0000000000003456] [Medline: 39432806]
